# Intervertebral Foramen Width Is an Important Factor in Deciding Additional Uncinate Process Resection in ACDF—a Retrospective Study

**DOI:** 10.3389/fsurg.2021.626344

**Published:** 2021-11-19

**Authors:** Baifeng Sun, Chen Xu, Yizhi Zhang, Shenshen Wu, Huiqiao Wu, Hao Zhang, Xiaolong Shen, Zifan Zhang, Wen Yuan, Yang Liu

**Affiliations:** ^1^Department of Spine Surgery, Shanghai Changzheng Hospital, Naval Medical University, Shanghai, China; ^2^Department of Trauma and Joint, People's Hospital of Liaoning Province, Shenyang, China

**Keywords:** uncovertebral joint, anterior cervical discectomy and fusion, uncinate process resection, cervical radiculopathy, intervertebral foramen decompression

## Abstract

**Background:** Anterior cervical discectomy and fusion (ACDF) has been established as a classic procedure for the management of cervical radiculopathy. However, it is unclear whether combined uncinate process resection (UPR) is necessary for treating cervical radiculopathy. Here, we investigated the clinical outcome of ACDF combined with UPR compared to ACDF alone to determine the necessity of UPR in treating cervical radiculopathy.

**Hypothesis:** Uncinate process resection may be necessary in certain patients along with ACDF to achieve better clinical outcomes of cervical radiculopathy.

**Patients and Methods:** Fifty-five patients underwent ACDF with UPR, and 126 patients without UPR were reviewed. The width and height of the intervertebral foramen were measured by 45° oblique X-rays. We also measured the Japanese Orthopedic Association (JOA) score and visual analog scale (VAS) score. C2–C7 Cobb angles were obtained from all patients pre- and post-operatively. Meanwhile, linear regression analysis was used to evaluate the relationship between the clinical outcomes and the intervertebral foramen width before surgery.

**Results:** Linear regression analysis indicated that the improvement in the JOA and VAS scores was irrelevant to both the pre-operative width of the intervertebral foramen (wIVF) and the height of the intervertebral foramen (hIVF) in the ACDF+UPR group. However, pre-operative wIVF was associated with post-operative JOA and VAS scores in the ACDF alone group. Those with pre-operative wIVF <3 mm in the ACDF group had the least improvement in post-operative clinical symptoms due to the change in wIVF (*P* > 0.05). The ACDF group whose wIVF was over 3 mm showed similar clinical outcomes to the ACDF + UPR group, and wIVF significantly increased post-operatively (*P* < 0.05). The fusion rate and C2–C7 Cobb angles did not show significant differences between the two groups (*P* > 0.05).

**Discussion:** Our current findings suggest that UPR should be considered when wIVF is <3 mm pre-operatively. However, there is no need to sacrifice the uncovertebral joint in ACDF when the pre-operative wIVF is over 3 mm.

**Level of Evidence:** Level III.

## Introduction

In cervical radiculopathy, uncovertebral osteophytes are one of the most common causes of nerve root compression (1, 2). As existing cervical nerve roots are anatomically close to the posterior aspect of the uncovertebral joint, the superior articular process, the ligamentum flavum, and the periradicular fibrous tissues may be involved in the compression of the nerve roots posteriorly (3, 4). However, anteriorly, only the uncovertebral joints are involved in nerve root compression, and an immunohistochemical and histological study supported that the uncovertebral joints could be a potential pain generator in cervical radiculopathy patients because osteophytes from the uncinate process (UP) can develop foraminal stenosis, resulting in cervical radiculopathy (5).

Anterior cervical discectomy and fusion (ACDF) has been established as a classic procedure for the management of cervical radiculopathy in patients who fail conservative treatment. Since the original ACDF procedure was reported, there has been no consensus on whether complete uncovertebral joint resection is needed when indirect decompression by distraction of the interbody space is performed during the ACDF procedure (6, 7). Some studies have indicated that symptomatic relief can be achieved through disc space distraction and resorption of the osteophytes after solid fusion (8). However, there is a paucity of research on the clinical outcomes after complete resection of uncovertebral osteophytes along with simultaneous ACDF, but some studies showed better neurological recovery and better final outcomes (9). Furthermore, uncinate process resection (UPR) might cause segment instability followed by decreased fusion rates and increased subsidence after ACDF (10–12). In addition, since the vertebral artery and nerve roots are anatomically close to the UP, the consequences could be catastrophic due to iatrogenic injury (13, 14). Due to the controversial role of uncovertebral joint resection, in this study, we sought to establish criteria to assess when UPR is necessary for treating patients with cervical radiculopathy.

## Materials and Methods

### Patients

**Figure 1:**
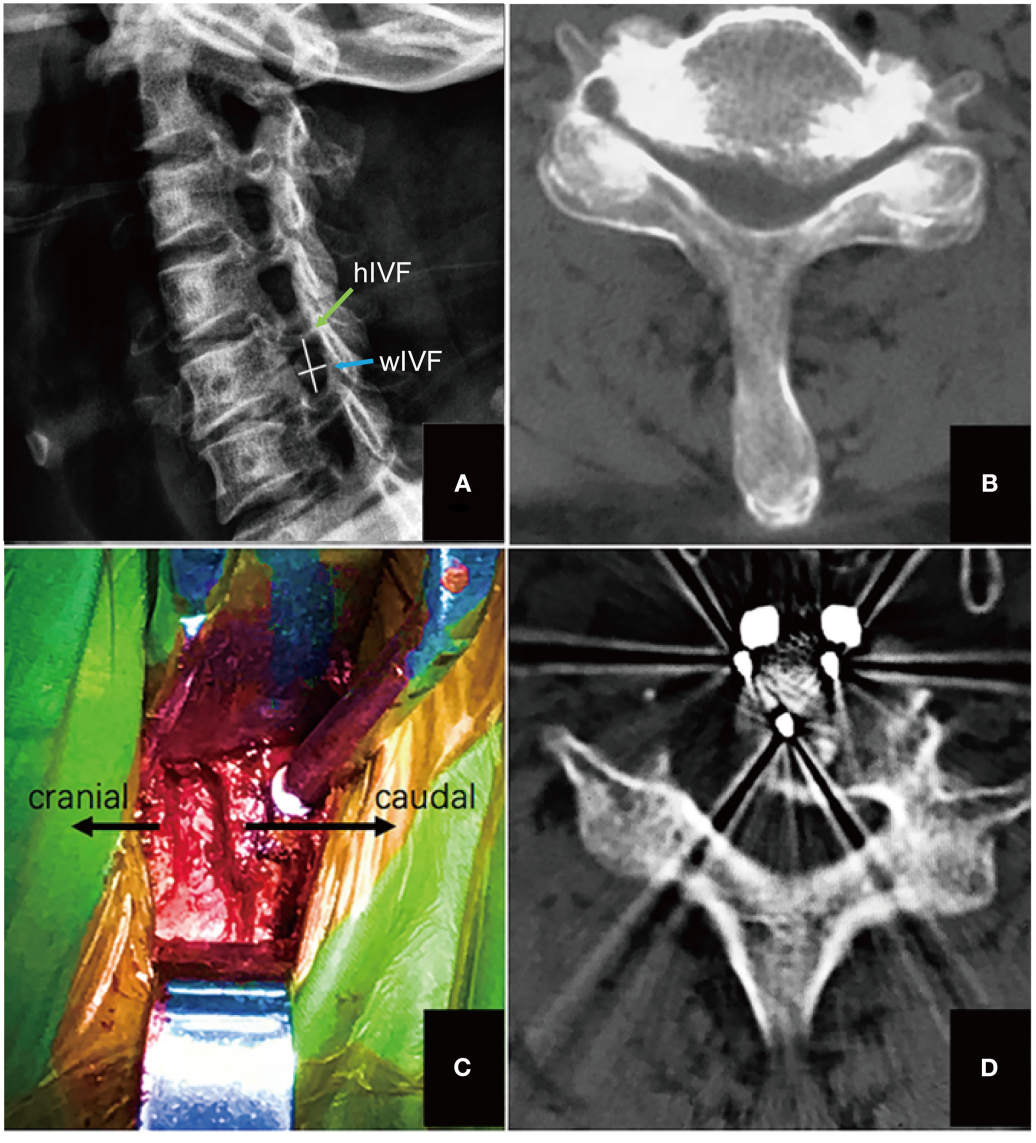
The process of performing UPR. **(A)** The width of intervertebral foramen (IVF) was measured by calculating the distance from the anterior medial point to the posterior medial point of the foramen (the transverse line) as width of IVF (wIVF), the height of IVF was measured by calculating the distance from the superior medial point to the inferior medial point of the foramen (the vertical line); **(B)** CT scan was used to confirm the hypertrophied uncinated process, meanwhile evaluating the anatomical relationship between vertebral artery and uncinated process; **(C)** the intraoperative view of surgical level after uncinated process was resected; **(D)** post-operative CT scan confirmed decompression of uncinated process osteophytes.

This study was approved by the ethics committee of Naval Medical University. Written informed consent was obtained from all participants, and the specific written informed consent for the publication of any potentially identifiable images or data included in this article of the enrolled patients were also obtained. From January 2016 to January 2017, cervical radiculopathy patients with radiological foraminal stenosis were invited to enroll in this study. The inclusion criteria were: (1) patients who were diagnosed with cervical radiculopathy. (2) There were fewer than 3 surgical segments from C3 to C6. (3) Intervertebral foraminal stenosis was demonstrated on both X-ray and CT scans. The exclusion criteria were: (1) patients who showed severe spinal cord compression [more than 50% (14)] on radiological examinations and/or displayed typical symptoms of cervical myelopathy, such as gait disturbance and weakness of the four extremities. (2) Patients who had implants of zero-profile or artificial cervical disks. (3) Patients who underwent bilateral UPR (Figure 1). (4) Patients who had cervical trauma, tumor or ossification of the posterior longitudinal ligament. Patients who met these criteria were asked whether they wanted to voluntarily participate in the blinded surgical grouping procedure; if not, standard ACDF was performed. Patients who voluntarily participated in the blinded surgical grouping procedure were divided into two groups according to a random number table: the ACDF + UPR group who underwent ACDF with the UPR procedure and the ACDF alone group. Based on these criteria, a total of 181 patients were included in our study; 55 patients were in the ACDF + UPR group, and 126 patients were in the ACDF-alone group. Plates and fusion material included Skyline (Johnson and Johnson Co., Depuy Spine Ltd., Ryhamn, MA), Elite plates (Medtronic Sofamor Danek Inc., Memphis, TN) and cages filled with autograft and allograft bone. All patients were suggested to wear a Philadelphia collar for 2 weeks after the operation.

### Radiographic Assessment

Pre-operative imaging studies assessed included anterior, neutral lateral, flexion/extension and 45° oblique radiographs, CT scans, and magnetic resonance imaging (MRI) of the cervical spine. MRI was acquired to evaluate disc decompression at the 6th month follow-up, and the fusion rate was observed by CT scans at the 12th month post-operatively. The width and height of the intervertebral foramen were measured by 45° oblique X-ray. The width was defined from the anterior medial zone of the superior vertebrae to the posterior medial zone of the inferior vertebra (Figure 1) (15).

### Clinical Outcomes

The JOA scoring system and VAS score were used to evaluate the improvements in neurologic function and arm pain. The nerve function recovery rate (RR) was calculated as RR= (post-operative JOA scores-pre-operative JOA scores)/(17-pre-operative JOA scores) ×100%, and the evaluation of pain release was measured by the recovery rate of VAS [(pre-operative VAS scores-post-operative VAS scores)/pre-operative VAS scores) ×100%]. In addition, the C2–C7 Cobb angles were obtained in all patients pre-operatively, at the 6th month post-operatively and at the 1-year follow-up. All parameters were measured by two different residents.

### Statistical Analysis

The SPSS software package [version 20.0 (IBM Corp. Armonk, New York, USA)] was used for statistical analysis. Each independent variable was compared between the two groups using the independent-sample Student's *t*-test for continuous variables. Linear regression was utilized to detect the relationship between the radiographic parameters and surgical outcomes. A value of *P* < 0.05 was accepted as significant.

## Results

**Figure 2:**
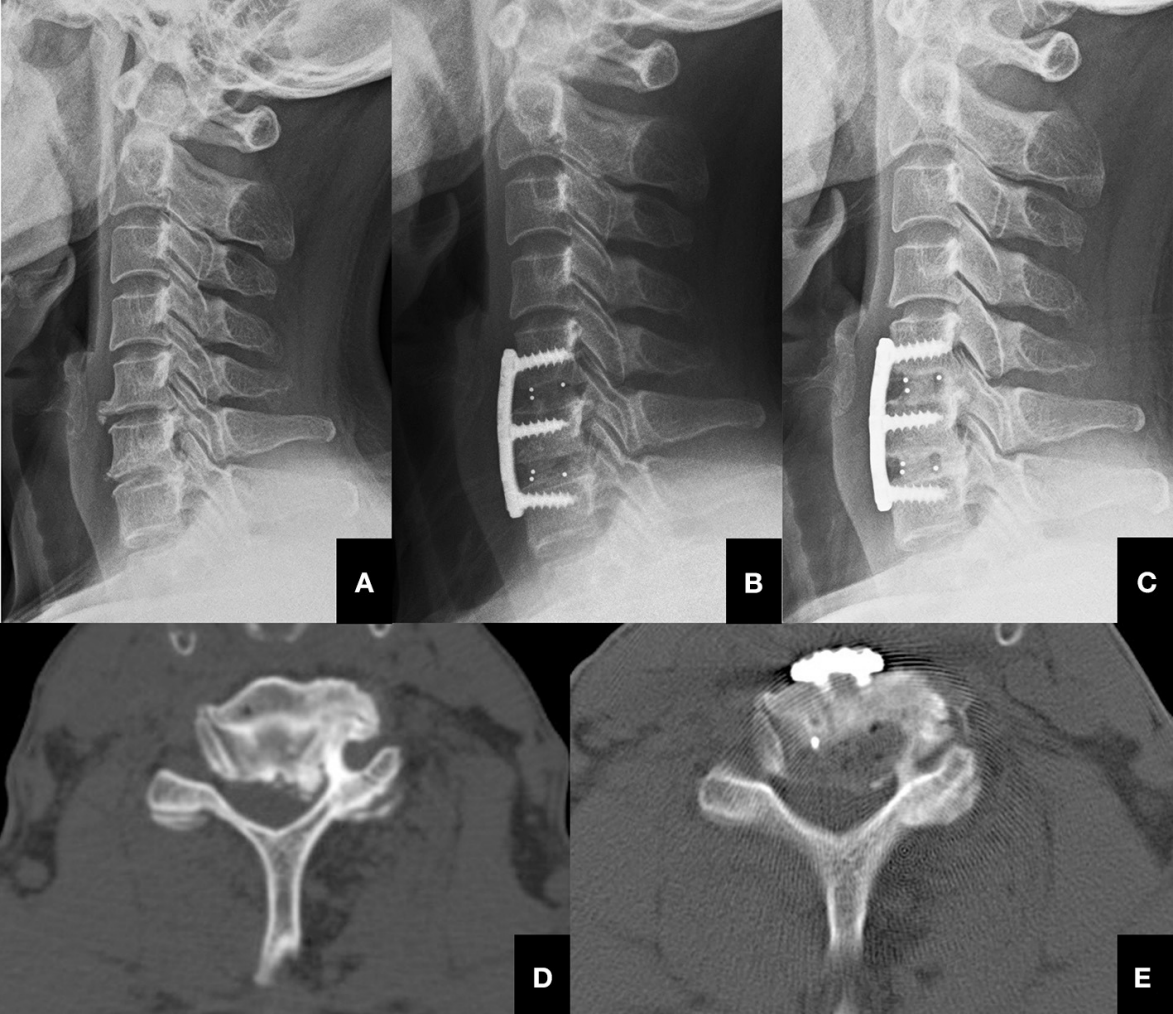
The process of performing UPR. **(A)** Typical lateral pre-operative X-ray image of a 56-year-old female patient with cervical radiculopathy ready to receive ACDF + UPR treatment. **(B)** Post-operative lateral X-ray image of the patient that received ACDF + UPR treatment. **(C)** One-year post-operative lateral X-ray image of the patient that received ACDF + UPR treatment, and showed spinal fusion was achieved and cervical alignment was sustained. (**D)** Pre-operative CT image showing severe hypertrophied left uncinate process of C5-C6 level. **(E)** Post-operative CT image showing the resected uncinate process of C5-C6 level.

Patients were followed up for a minimum of 1 year (range from 12 to 30 months). Sex, age, body mass index (BMI), and operation level were not different between the 2 groups (Table 1). MRI demonstrated that all patients achieved sufficient decompression of disc space at the 12-month follow-up. In patients treated with ACDF + UPR, 94.5% patients (52 out of 55) achieved spinal fusion at 1-year follow-up (Figure 2), and 91.3% patients (115 out of 126) achieved spinal fusion at 1-year follow-up. All patients received neurological recovery at 1-year follow-up. Persistent post-operative axial pain (post-operative axial pain sustained more than 6 months) was found in 1 patient in ACDF + UPR group, and 6 patients in ACDF alone group.

**Table 1:** Comparison of study patient demographics.

	**ACDF + UPR**	**ACDF**	***P*-value**
Gender (Male: Female)	30:25	67:59	0.38
Age (years)	58.2 ± 10.2	58.8 ± 10.9	0.52
BMI (kg/m^2^)	24.63 ± 9.25	24.38 ± 8.82	0.46
1-level	32	60	
C3-4	3	7	
C4-5	13	20	
C5-6	16	33	
2-level	23	66	
C3-5	3	12	
C4-6	20	54	

*ACDF, anterior cervical discectomy and fusion; UPR, uncinate process resection; BMI, body mass index. P < 0.05 is considered as significant*.

**Figure 3:**
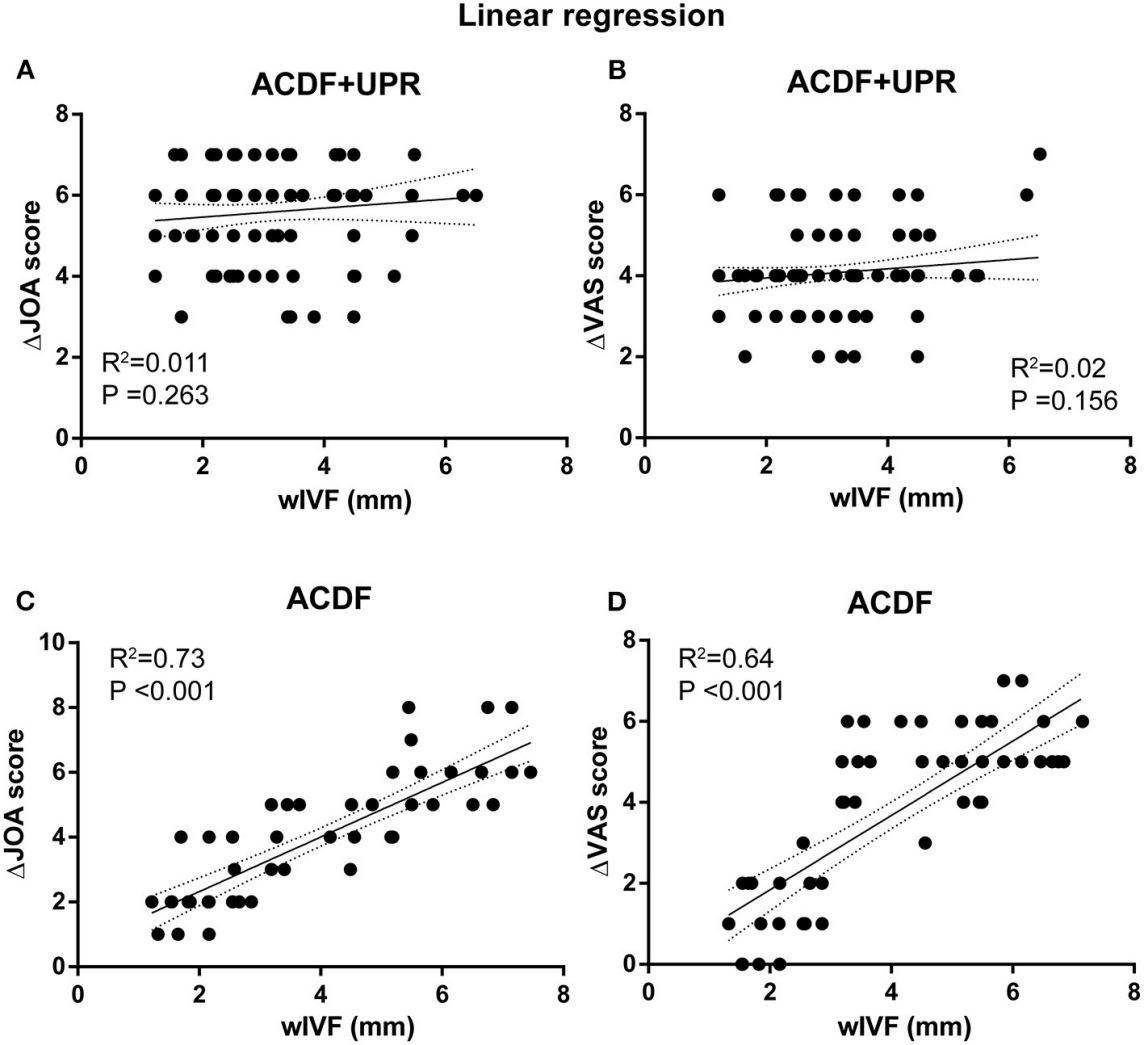
Linear regression plots of VAS and JOA score in ACDF + UPR or ACDF alone. Linear regression analysis of VAS and JOA score in ACDF + UPR or ACDF alone patients. Clinical improvement was calculated as (post-operation value)—(pre-operation value) for JOA score (**A,C**) and shown as ΔJOA score; (pre-operation value)—(post-operation value) for VAS (**B,D**) score, and shown as ΔVAS score. *P* < 0.01 is concerned as significant. While only ACDF groups showed significant correlation of wIVF with clinical improvement (**C,D**).

The change of width and height of intervertebral foramen (IVF) in different treatment groups was assessed first, and we found that the width of IVF (wIVF) was significantly increased compared to that of height of IVF (hIVF, Table 2). Since wIVF is significantly affected after both treatments, we performed linear regression analysis to study the relevance of wIVF with clinical outcome. The results demonstrated that the improvement of JOA and VAS scores was irrelevant to the pre-operative wIVF in ACDF + UPR but was associated with pre-operative wIVF in ACDF alone (Figure 3). To further identify this phenomenon, we divided these ACDF-alone patients according to their wIVF width and evaluated the differences in JOA and VAS scores between each subgroup. We found that subgroups with wIVF <3 or >4 mm showed no differences in their JOA and VAS scores (Table 3). However, when comparing the 2~ with 3~ mm subgroups, the patients had a significant difference in JOA and VAS improvement (Table 3). Such data indicate that 3 mm in wIVF may be a critical factor for the outcome of ACDF alone. However, this factor does not affect the outcome of ACDF + UPR patients.

**Table 2:** The change of width and height of intervertebral foramen in different treatment groups.

**Parameters**	**Groups**	**Pre-operation**	**Post-operation**	***P*-value**
wIVF	ACDF	3.21 ± 1.09	4.38 ± 1.16	0.03
	ACDF + UPR	3.39 ± 1.14	4.36 ± 1.18	0.02
hIVF	ACDF	10.31 ± 1.58	10.83 ± 2.04	0.33
	ACDF + UPR	10.26 ± 1.60	10.95 ± 1.94	0.21

*wIVF, the width of intervertebral foramen; hIVF, the height of intervertebral foramen. Post-operative parameters were compared to the pre-operative ones using two-sided t-test. P < 0.05 is considered as significant*.

**Table 3:** The differences of JOA and VAS score between each wIVF subgroup of ACDF alone patients.

**wIVF (mm)**	**JOA**		**VAS**	
	***n***	***P*-value**	***n***	***P*-value**
1~	19	0.435	19	0.518
2~	32	<0.001	32	<0.001
3~	16	0.618	16	0.421
4~	23	0.07	23	0.273
5~	21	0.876	21	0.785
6~	9	0.898	9	0.908
7~	6	–	6	–

*Each subgroup is compared to the next subgroup using two-sided t test. wIVF, width of intervertebral foramen; JOA, Japanese orthopedic association; VAS, visual analog scale. P < 0.05 is considered as significant*.

To obtain more information about this factor, we divided the patients into a wIVF <3 mm group and a wIVF ≥ 3 mm group and analyzed the clinical outcomes at different time points ranging from pre-operation and post-operation to the 1-year follow-up. Although the recovery rate showed no significant differences between the group (Table 4), patients with pre-operative wIVF <3 mm in ACDF alone showed less improvement in JOA and VAS scores (Table 5). In further research, we found that the mean pre-operative wIVF was 2.01 ± 1.09 mm, and the post-operative wIVF was 2.38 ± 0.86 mm in those patients whose wIVF was within 3 mm in the ACDF alone group, which showed no significant difference (*P* > 0.05). For other patients in the ACDF alone group whose wIVF was over 3 mm, the wIVF significantly increased from 3.29 ± 1.14 mm pre-operatively to 5.36 ± 1.18 mm post-operatively (*P* < 0.05).

**Table 4:** Comparison of the recovery rate of ACDF with UPR or non-UPR technique.

	**Recovery rate**		***P*-value**
	**ACDF + UPR**	**ACDF**	
JOA	78.26 ± 10.17%	73.26 ± 11.35%	0.58
VAS	86.64 ± 9.62%	81.14 ± 10.72%	0.21

*ACDF, anterior cervical discectomy and fusion; UPR, uncinate process resection; JOA, Japanese orthopedic association; VAS, visual analog scale. P < 0.05 is considered as significant*.

**Table 5:** Comparison of clinical outcomes in four groups.

		**ACDF** **+** **UPR**		**ACDF**		***P*-value**
		**<3 mm**	**≥3 mm**	**<3 mm**	**≥3 mm**	
Fusion rate	1-year follow up	90.00%	92.00%	92.16%	94.67%	0.35
JOA score	Pre-operative	9.88 ± 1.59	10.27 ± 1.69	10.02 ± 1.71	10.35 ± 1.56	0.31
	Post-operative	15.47 ± 1.63	15.28 ± 1.70	13.37 ± 1.75	15.58 ± 1.65	0.03
	Post-operative RR	78.26 ± 10.32%	76.42 ± 11.25%	50.49 ± 11.17%	79.37 ± 11.62%	0.01
	1-year follow-up	16.27 ± 0.83	16.02 ± 0.87	14.57 ± 0.95	16.32 ± 0.71	0.01
	1-year RR	90.26 ± 5.12%	87.63 ± 6.07%	65.57 ± 6.52%	90.87 ± 5.35%	0.01
VAS score	Pre-operative	6.26 ± 1.05	5.32 ± 0.91	6.14 ± 1.07	5.24 ± 1.13	0.16
	Post-operative	0.86 ± 0.49	0.78 ± 0.37	3.38 ± 1.23	0.62 ± 0.33	0.01
	Post-operative RR	85.72 ± 8.13%	87.16 ± 8.07%	46.5 ± 9.63%	89.25 ± 7.68%	0.01
	1-year follow-up	0.90 ± 0.31	0.76 ± 0.29	1.32 ± 0.43	0.79 ± 0.25	0.01
	1-year RR	90.32 ± 5.52%	91.69 ± 5.32%	78.50 ± 6.23%	92.55 ± 5.13%	0.01
C2-C7 Cobb angles	Pre-operative	11.21 ± 4.84	11.32 ± 4.92	11.15 ± 5.02	11.38 ± 5.15	0.36
	Post-operative	15.17 ± 5.06	15.36 ± 4.98	16.90 ± 5.23	15.76 ± 5.42	0.28
	1-year follow-up	17.56 ± 5.12	16.71 ± 5.08	17.08 ± 5.35	16.98 ± 5.19	0.31

*ACDF, anterior cervical discectomy and fusion; UPR, uncinate process resection; JOA, Japanese orthopedic association; VAS, visual analog scale; RR, Recovery rate. P <0.05 is considered as significant*.

The fusion rate also showed no significant difference (*P* > 0.05) between groups, which was confirmed by CT scans at 12 months post-operatively. Meanwhile, graft subsidence was not observed at the final follow-up by CT scan, and the C2–C7 Cobb angles were also insignificant between the groups (*P* > 0.05).

## Discussion

Foraminal stenosis due to osteophyte accumulation or hypertrophy of the uncovertebral joints can result in impingement on nerve roots as they exit behind the uncovertebral joint. Previous studies have confirmed that ACDF is a good surgical option for the treatment of patients with neck pain and cervical radiculopathy, but the role of direct uncovertebral joint decompression has been somewhat controversial (16). Theoretically, if ACDF alone can achieve satisfactory clinical outcomes after thorough removal of the disc tissue, it is obviously unnecessary to remove the uncinate process.

In the process of UPR, the anterior structure of the intervertebral foramen is mainly destroyed. Therefore, the relationship between pre-operative wIVF and clinical outcomes can potentially provide a theoretical basis for the timing of UPR. In our study, linear regression analysis confirmed that the improvement of JOA and VAS scores was not significantly associated with pre-operative wIVF in the ACDF + UPR group, and both JOA and VAS scores were improved significantly, even if the pre-operative intervertebral foramen stenosis was very severe. In the ACDF alone group, linear regression demonstrated that the improvement in the JOA and VAS scores was relevant to the pre-operative wIVF. In further research, we found that the patients with pre-operative wIVF <3 mm in Group B showed the worst improvement post-operatively. On the other hand, patients with a pre-operative wIVF <3 mm in the ACDF+UPR group showed significant improvement in their JOA and VAS scores. Therefore, we believe that for patients with severe stenosis of the intervertebral foramen <3 mm, ACDF+UPR should be considered (Figure 3, Table 3). We also found that, regardless of whether the UPR was performed, the improvement in the JOA and VAS scores was significant for those patients with pre-operative wIVF >3 mm in both groups. Thus, it is unnecessary to perform UPR for these patients, and ACDF alone can achieve satisfactory clinical outcomes.

In most cases, the narrowing of IVF could be caused by anterior osteophytes, the posterior longitudinal ligament and other soft tissue restraints combined with simultaneous subluxation. Adequate disc height distraction and restoration of the sagittal alignment can sometimes increase both the height and width of IVF. The height of the intervertebral foramen was normally about 8–11 mm, whereas the nerve root was only <3 mm. Thus, the cervical nerve root only consumes <50% of the available height of the intervertebral foramen in the neutral position. But the width of the intervertebral foramen can be <3 mm wide in some severe cases, which may be the main cause that resulted in radiculopathy symptoms. Specifically, C4–C6 nerve roots were reported to be approximately 3 mm in width, and the widths of the dorsal ganglions were generally over 3 mm (17–19). Thus, a heightened disc space and a restored sagittal alignment are not enough to achieve adequate decompression of the wIVF, and the wIVF should be at least 3 mm to avoid nerve root compression after the surgery. Although the bony width was normally found to be around 4 mm after surgery, the actual wIVF might be smaller than this value due to hypertrophied tissues that reside in the foramen, which meant that nerve roots and dorsal ganglions could still be compressed even after disc space distraction and restoration of the sagittal alignment. This may explain why the patients with wIVF <3 mm in the ACDF group had the least improvement of symptoms.

Since single-side vertebral articulation can contribute up to more than 60% of the stability of the spinal motion segment in extension (20, 21), some researchers were concerned that the cervical stability of uncovertebral joints could be relevant in the presence of a solid fusion. Thus, instability and motion at the graft site could reduce the ultimate fusion rate (22). Since three- and four-level ACDF appears to have a high incidence of pseudarthrosis, we only evaluated patients whose surgical segments were within 2 levels (23). In our research, there was no significant difference in the fusion rate between the 2 groups, and we found that most patients received ACDF+UPR achieved solid fusion at 1-year follow-up (Figure 2); therefore, we proved that the unilateral UPR did not affect the rate of fusion. Nevertheless, in our opinion, UPR should only be performed with caution. It may result in a prolonged surgical operation time compared with ACDF alone. Furthermore, vertebral artery injury is a catastrophic complication that can occur during UPR at a high rate, although the reported rate of vertebral artery injury is quite low, ranging from 0.3 to 0.5% during ACDF (24, 25). It has been proven that bilateral UPR is correlated with subsidence after ACDF [12]. In this study, probably due to preservation of the contralateral UP, subsidence of the graft was not observed in either group by CT scans. In theory, UP plays an important role in maintaining cervical stability, so unilateral UP may result in a better circumstance for union than bilateral UPR.

Here, we used the 45° oblique cervical spine X-ray to measure the width and height of intervertebral foramen, because oblique X-ray is the most convenient and safe way to achieve the view of the intervertebral foramen. A previous study demonstrated a 45° oblique view of the cervical spine to demonstrate the neural foramen on plain radiograph examination (26). Besides, compared with CT scan, oblique X-ray can reduce the harm of radiation, also obtain similar measure results (27). Nevertheless, high resolution CT scan and three-dimensional reconstruction is best for the complete measurement of the intervertebral foramen. Another limitation is that the sample size in the ACDF+UPR groups is relatively small, and we will keep collecting the related cases and data for further in-depth studies.

In summary, although ACDF combined with unilateral UPR could achieve excellent clinical outcomes, it should not be performed routinely. When wIVF is over 3 mm pre-operatively, disc space distraction and restoration of the sagittal alignment can facilitate adequate decompression post-operatively. However, UPR should be considered when wIVF is <3 mm pre-operatively.

## Conclusions

Our current findings suggest that UPR should be considered when wIVF is <3 mm pre-operatively. However, there is no need to sacrifice the uncovertebral joint in ACDF when pre-operative wIVF is over 3 mm.

## Data Availability Statement

The raw data supporting the conclusions of this article will be made available by the authors, without undue reservation.

## Ethics Statement

The studies involving human participants were reviewed and approved by Changzheng Ethics Committee. The Ethics Committee waived the requirement of written informed consent for participation.

## Author Contributions

This study was designed by YL and WY and drafted by BS and CX. HZ, SW, and HW contributed to collecting data. YZ and ZZ conducted the statistics analysis. All authors contributed to the article and approved the submitted version.

## Funding

This research was supported by grants from the National Natural Science Foundation of China (81772392, 81972090, 82172470, 81772376, and 82072471), Shanghai Science & Technology Commission Rising-Star Program (20QA1409200) and Shanghai Education Development Foundation and Shanghai Municipal Education Commission Rising Stars of Medical Talent Youth Development Program, Shanghai Changzheng Hospital Medical Service Innovation Project (2020CZWJFW15) and High-Quality Research Cultivating Project (2020YCGPZ-207).

## Conflict of Interest

The authors declare that the research was conducted in the absence of any commercial or financial relationships that could be construed as a potential conflict of interest.

## Publisher's Note

All claims expressed in this article are solely those of the authors and do not necessarily represent those of their affiliated organizations, or those of the publisher, the editors and the reviewers. Any product that may be evaluated in this article, or claim that may be made by its manufacturer, is not guaranteed or endorsed by the publisher.

## Abbreviations

ACDF: anterior cervical discectomy and fusion
UPR: uncinate process resection
JOA: Japanese Orthopedic Association
IR: Improvement rate
VAS: visual analog scale
ROM: range of motion
wIVF: width of intervertebral foramen
UP: uncinate process
RR: recovery rate.
